# Individual Differences in Mental Accounting

**DOI:** 10.3389/fpsyg.2019.02866

**Published:** 2019-12-18

**Authors:** Stephan Muehlbacher, Erich Kirchler

**Affiliations:** Faculty of Psychology, University of Vienna, Vienna, Austria

**Keywords:** mental accounting, theater-ticket problem, income source, conscientiousness, impulsivity

## Abstract

Individual differences in mental accounting have rarely been studied, and empirical evidence regarding the relation between mental accounting and personality characteristics is scarce. The present paper reports three studies applying a Likert-type scale to assess the extent individuals engage in mental accounting practices. In each study, the five items of the measure loaded on a single dimension and had acceptable reliability, with a Cronbach’s α between 0.72 and 0.77. Study 1 (*N* = 165) regards the mental processing of prior losses in the theater-ticket problem (Tversky and Kahneman, 1981). Study 2 (*N* = 114) is based on prior work on income source effects (Fogel, 1997) and analyzes mental accounting of prior gains. In both studies, individual differences in mental accounting moderated the effects of the experimental treatments. In an explorative survey conducted for Study 3 (*N* = 373), the extent of engaging in mental accounting was found to be positively correlated with being female, with conscientiousness, and financial literacy, and negatively related with education and non-planning impulsivity. Identification of individual differences and their correlates adds to existing evidence for some of the core assumptions of mental accounting theory. A practical implication of the findings is that providers of financial services must take individual differences into account when designing trainings and supportive tools for money management.

## Introduction

Mental accounting theory (Thaler, 1985, 1999) has been studied for decades. First empirical evidence suggesting that individuals manage their budget and expenses by “psychological accounting” was reported from the well-known theater-ticket study by Tversky and Kahneman (1981, p. 457). They asked respondents from two samples whether they would pay another $10 for a theater ticket after noticing either the loss of a $10 bill or the loss of a presale theater ticket of similar value. Those reading about the loss of a dollar bill were more willing to buy another ticket (88% of *n* = 183) than respondents reading about losing the presale ticket (46% of *n* = 200). Losses occurring in a specific spending category (e.g., expenses for entertainment) as in the case of a lost ticket affect further spending in the same category more than losses without a “label” as in the case of the lost dollar bill. This observation violates the economic principle of fungibility – that money has no “labels” – and suggests that spending (and other financial) decisions are affected by categorization processes and the resulting mental organization of the budget (Henderson and Peterson, 1992). The idea of mental accounting was elaborated by Thaler (1985, 1999) to a broad descriptive theory of consumer choice. Since then, it has been applied to a wide range of financial behaviors as, for instance, stock market investments (Grinblatt and Han, 2005), spending and saving decisions (Shefrin and Thaler, 1988), usage of credit cards (Prelec and Simester, 2000), gambling (Cowley, 2008), and taxpayers’ compliance (Muehlbacher and Kirchler, 2013). For a recent review of the literature, see Zhang and Sussman (2018).

Most empirical research on mental accounting studies its general effects on behavior. Only a few attempts were made to explore individual differences in mental accounting, their causes, and their consequences for behavior. To fill this gap, the present paper attempts to assess such interindividual variation, studies its consequences for choice, and explores its correlates. We will report results from two experiments demonstrating the moderating role of individual differences for well-documented behavioral effects of mental accounting and results from a survey exploring the psychological constructs and socio-demographical characteristics related to mental accounting. Study 1 focuses on mental accounting of losses and replicates the theater-ticket study described above (Tversky and Kahneman, 1981). Study 2 regards mental accounting of gains and replicates a study on income source effects on spending (Fogel, 1997). In both studies, our measure for individual differences to engage in mental accounting moderated the effects of the experimental manipulation. The explorative survey conducted for Study 3 revealed that the extent to engage in mental accounting correlates with conscientiousness, impulsivity, financial literacy, education, and gender. Before the three studies are reported, we will briefly review the core aspects of mental accounting theory, prior attempts to measure individual differences, other psychological dispositions related to economic decisions, and existing evidence for correlates of mental accounting.

## Mental Accounting Theory

Thaler (1999) defined mental accounting as “[…] the set of cognitive operations used by individuals and households to organize, evaluate, and keep track of financial activities” (p. 183). Mental accounting processes are assumed to serve (at least) three purposes: to simplify decisions, to keep self-control when facing tempting consumption opportunities, and to maximize hedonic pleasure from decision outcomes (Antonides and Ranyard, 2017; Zhang and Sussman, 2018). In his 1999 review, Thaler summarized prior studies on mental accounting. One stream of research concerns perceptions and evaluations of outcomes. For instance, two separate gains are often perceived as larger than their integrated value (e.g., Thaler and Johnson, 1990; Linville and Fischer, 1991), and sometimes, it matters more for consumers whether a transaction is considered advantageous and fair than the value of the acquired product or service itself (e.g., Thaler, 1985; Muehlbacher et al., 2011). Another stream of research described in Thaler’s (1999) review regards temporal aspects of mental accounting. Account balances may be checked seldom or often, which affects the evaluation of the budget and, consequently, its spending (Heath and Soll, 1996). For instance, when most bills need to be paid monthly and accounts are evaluated with according frequency, receiving smaller amounts of income every month affects spending more than a larger sum received once a year (Chambers and Spencer, 2008). The third – and probably most prominent – stream of mental accounting research concerns the budgeting process. As explicated in the introductory section, individuals tend to label and categorize money and its uses. They track their financial activities by grouping them into categories (e.g., housing, food, and entertainment) and restrict expenses in each category to a predefined budget (Heath and Soll, 1996; Thaler, 1999). Expenses may be assigned to the same category, for instance, because they are made to pursue the same goal (e.g., nutrition and entertainment), are of similar magnitude (e.g., things to do with €10 and exceptionally large sums), share the same mode of payment (e.g., credit card and cash), or occur at the same location (e.g., electronics store, drug store, and vacation resort). The rationale of organizing expenses in mental accounts is to reduce the computational costs for spending decisions, to facilitate self-control, and to prevent overspending (Heath and Soll, 1996). Put in other words, consumers engage in mental accounting “to keep track of expenses for things we like, and to save money for things we don’t like but still have to pay” (Muehlbacher and Kirchler, 2013, p. 414).

## Assessing Individual Differences in Mental Accounting

Mental accounting is typically studied in lab experiments designed to create decision situations for which the theory predicts choices deviating from the assumptions of traditional economics. In tradition of Tversky and Kahneman’s (1981) theater-ticket study, sources or labels of income are manipulated, and the effects of the experimental treatment on expenses are analyzed (e.g., Heath and Soll, 1996; Fogel, 1997; Cheema and Soman, 2006). Only a few studies applied different methods such as interviews (e.g., Adams and Webley, 2001; Ashby and Webley, 2008a, b; Muehlbacher and Kirchler, 2013) or quasi-experiments in the field (e.g., Jackson et al., 2005; Helion and Gilovich, 2014). Surprisingly, in particular, questionnaire surveys are largely underrepresented in mental accounting literature (the few existing survey studies focus predominantly on the questions whether individuals restrict themselves to predefined budgets and how longsighted their financial planning horizon is; Zhang and Sussman, 2018), although questionnaires are a cost-efficient tool for data collection and allow us to survey target populations that normally would not participate in lab experiments. A consequence of the lack of survey research is that attempts to measure mental accounting practices – in contrast to experimentally inducing a situation that leads to one or another practice – are scarce. Accordingly, little is known about individual differences in how mental accounting is applied and about correlates of the variance.

The first endeavor to measure mental accounting practices was conducted by Soman (2001). He applied two Likert-type scales with eight items each to analyze the difference between mental accounting of monetary and time costs. However, whether participants’ responses on these scales were related with any other variables was not tested in this study. The next attempt was published a decade later (Antonides et al., 2011). The scale applied in this survey study consists of four items regarding specific aspects of the mental budgeting process (reserving money for different expenses, sticking to a predefined budget, economizing on other expenses after a large expenditure, and economizing in the next month after spending more than normal in the current month). The responses to the items indicate the degree to which participants pursue the four mentioned strategies. In another survey, mental accounting practices regarding tax liabilities – a very specific detail in the mental organization of budgets – were measured using a 10-item scale (Muehlbacher and Kirchler, 2013). On the basis of the protocols of preceding interviews with self-employed taxpayers, this Likert-type scale was developed to differentiate between mental segregation and integration of income and the tax due, i.e., whether respondents tended to keep a mental account dedicated specifically to the tax liability. Adapted and revised versions of this scale were used in another survey comparing income tax with VAT compliance (Olsen et al., 2019) and in a lab experiment on tax compliance (Muehlbacher et al., 2017).

Besides the above-mentioned Likert-type measures for individual differences in mental accounting, prior research has also applied few completely different methods. For instance, participants in an experiment were provided with a hypothetical list of expenses that should be labeled and categorized into a self-constructed accounting system. The number of accounts that were set-up was interpreted as a measure reflecting individuals’ mental accounting practices. It was analyzed as one of the dependent variables of the experimental manipulation. However, potential correlations with other variables were not explored in this study (Cheema and Soman, 2006). In an interview study on money management of taxpayers, the number of statements expressing two opposed principles for mentally processing the tax due (integration vs. segregation with net income) served as a measure for the preference in applying mental accounting when administering tax payments. The index was related to the point of time when the interviewees brought up taxes as an issue while talking about their money management (Muehlbacher and Kirchler, 2013).

In sum, only little research has tried to measure the individual differences in engaging in various mental accounting practices, i.e., treating mental accounting as a trait instead of analyzing its general behavioral effects in reaction to a specific situation. Some of the above-mentioned alternative approaches may be creative but are far more complicated to implement than common Likert-type scales. Hence, for the studies presented here, we applied a simple measurement scale to demonstrate the role of individual differences in mental accounting and to explore for its correlates.

## Other Psychological Dispositions Related to Financial Behavior

Mental accounting is defined as a theory about the cognitive processes accompanying and affecting financial behavior. However, in research practice, often a broader view seems to be taken about what mental accounting is, and sometimes, the boundaries to actual financial behavior and to other theoretical concepts applied in money management literature are blurry. McNair and Crozier (2017) provided a comprehensive review regarding psychological dispositions that affect economic decisions. Some of the concepts discussed therein seem to be related with mental accounting and overlap with certain aspects of the theory. The first category of dispositions that they distinguish is financial attitudes. For instance, money attitudes encompass individual beliefs and ethics regarding the use and purpose of money such as how much money is seen as a source of power, as the source of good and evil, or as a tool to demonstrate generosity. In addition, attitudes toward credit and debt and toward materialistic values fall into this category. Moreover, compulsive and impulsive buying are often conceptualized as the behavioral outcomes of financial attitudes. The second category of dispositions regards temporal aspects of financial behavior. Individuals differ, for instance, in the time perspective that they typically adopt, i.e., whether they are oriented toward the past, present, or future, and in the extent to which spending of resources is planned. Further, the (in)ability to delay gratifications and how much future rewards are discounted varies between individuals. The third category concerns pragmatic dispositions affecting financial decisions. It covers, for instance, the perceived locus of control regarding financial outcomes (i.e., whether these are attributed to internal factors such as effort and skill or to external factors such as luck or social context) and skills and behaviors regarding the management of money, such as preparing a budget, checking bank accounts, and keeping overview of finances. In contrast to mental accounting, the focus here is on real behavior rather than the accompanying cognitive processes. However, at least at the level of measurement, the two concepts seem to overlap. Another disposition in the third category regards financial knowledge, with financial literacy being probably the most prominent concept in this category. As Hastings et al. (2013, p. 349) summarized, definitions of financial literacy vary and sometimes encompass not only the knowledge of financial products, the knowledge of financial concepts, and having appropriate mathematical skills, but they are also to be engaged in certain activities such as financial planning. Again, the latter aspect seems to overlap with definitions of mental accounting and money management in general. The most widespread method for assessing financial literacy (the “Big Five” questions, which were also applied in the survey of Study 3), however, focuses solely on knowledge and understanding of financial concepts.

## Correlates of Mental Accounting

As most prior research deals with the general effects of mental accounting, only little is known about correlates of the mental accounting processes apart from its behavioral outcomes. Without citing empirical evidence for his assumption, Thaler (1999) stated in his review the following:

Of course, there is considerable variation among households in how explicit the budgeting process is. As a rule, the tighter the budget, the more explicit are the budgeting rules, both in households and organizations. Families living near the poverty level use strict, explicit budgets; in wealthy families budgets are both less binding and less well defined. (p. 193)

However, such variations, their causes, and their consequences have remained largely unexplored. Empirical findings from such research are summarized in the following.

The first group of variables that has been addressed in prior work can be regarded as the consequences of different mental practices. The extent to which mental accounting is pursued was found to be positively related to having overview of expenses and to money management (Antonides et al., 2011). A measure for mental accounting of the tax due was linked to tax compliance (Muehlbacher and Kirchler, 2013; Muehlbacher et al., 2017), although in a follow-up study, the correlation with compliance was found only for some of the measure’s subscales (Olsen et al., 2019).

The second group of variables concerns respondents’ socio-demographic characteristics. In two studies, females were more likely to engage in the practices examined by the respective mental accounting measure (Antonides et al., 2011; Muehlbacher et al., 2017), but two other studies found no gender effect (Muehlbacher and Kirchler, 2013; Olsen et al., 2019). In addition, for age, some studies report a positive correlation (Muehlbacher and Kirchler, 2013; Muehlbacher et al., 2017), whereas others observed no relation (Antonides et al., 2011; Olsen et al., 2019). Wealth was found to be negatively related to mental accounting (Antonides et al., 2011), but income, by contrast, was found to be positively related (Muehlbacher and Kirchler, 2013; Olsen et al., 2019), and financial scarcity was found to be negatively related (Olsen et al., 2019). Financial scarcity was also found to be a moderator of several mental accounting phenomena in a series of experimental studies. The results show that, for instance, transaction utility and proportional thinking in the evaluation of discounts affect consumers with scarce resources to a lesser extent than high income earners. However, for the theater-ticket problem, no moderation effect of scarcity was found (Shah et al., 2015). In a survey study, higher education was associated with engaging less in mental accounting practices (Antonides et al., 2011). In line with this result are findings from a laboratory experiment, which showed that students with lower grades in math exams are more prone to labeling effects of income and thus more often violate the economic notion of fungibility (Abeler and Marklein, 2016). In a survey among self-employed entrepreneurs, the number of employees was negatively related to mental accounting (Muehlbacher and Kirchler, 2013).

The third group of potential correlates of mental accounting concerns respondents’ attitudes, knowledge, skills, and personality characteristics. Keeping a mental account dedicated specifically to the tax due was positively related to attitudes toward paying taxes (Muehlbacher and Kirchler, 2013; Muehlbacher et al., 2017; Olsen et al., 2019). Further, tax knowledge (Olsen et al., 2019) and self-assessed financial knowledge (Antonides et al., 2011) were positively related, whereas financial literacy – a more objective measure of financial knowledge – was not related (Olsen et al., 2019).

Regarding personality characteristics, a time orientation with focus on the near future was found to be negatively related (Antonides et al., 2011; Olsen et al., 2019) and long-term orientation to be positively related (Antonides et al., 2011) with mental accounting. A psychological construct closely related to time orientation is impulsivity. It is defined as a personality trait characterized by rapid, unplanned actions without thinking about potential negative consequences. The three subtypes, namely, non-planning impulsivity, motor impulsivity, and attentional impulsivity, are often differentiated in the literature (Meule et al., 2011). Since mental accounting is (also) a theory about self-control, it may be hypothesized that impulsive individuals with low self-control are less likely to engage in mental accounting (Antonides and Ranyard, 2017). So far, the only evidence for this assumption has been reported from a survey on mental accounting of the tax due (Olsen et al., 2019), which was conducted after the data analysis for the present paper and hence already incorporated some of its conclusions.

Conceptualizing mental accounting as a trait brings up the question whether and how it is related to other psychological traits such as the five characteristics described in the most prominent taxonomy of personality. The so-called Big Five (e.g., McAdams and Pals, 2006) include the traits extraversion or surgency (talkative, assertive, and energetic), agreeableness (good-natured, cooperative, and trustful), conscientiousness (orderly, responsible, and dependable), emotional stability vs. neuroticism (calm, not neurotic, and not easily upset), and intellect or openness (intellectual, imaginative, and independent-minded; John and Srivastava, 1999, p. 105).^1^ Although these traits have not yet been linked to mental accounting theory, their concepts have already been applied in many other areas of decision research as, for instance, in explaining risk taking (Nicholson et al., 2005; Pinjisakikool, 2018), framing effects (Levin et al., 2002), anchoring effects (McElroy and Dowd, 2007), overconfidence (Schaefer et al., 2004), discounting of delayed rewards (Hirsh et al., 2008), and decisions under social and time pressure (Byrne et al., 2015). The traits extraversion, agreeableness, neuroticism, and openness were found to be associated with an intuitive decision style, and emotional stability and conscientiousness were associated with a deliberate style (Betsch, 2004). On the basis of these results, it can be hypothesized that if mental accounting is the byproduct of a deliberate decision process, it should be associated with emotional stability and conscientiousness. On the other hand, if mental accounting leads to more intuitive decisions, for instance, by its function to simplify decisions, this would mean that it should correlate with extraversion, agreeableness, neuroticism, and openness.

In addition, research on money management has analyzed the role of the Big Five of personality. For instance, although neuroticism and extraversion were found to be related with saving less and having more debt (Nyhus and Webley, 2001), openness and conscientiousness were associated with more savings and more long-term investments (Brandstätter, 1996, as cited in Pinjisakikool, 2018; Mayfield et al., 2008). Moreover, highly conscientious individuals manage their finances more carefully, which was attributed to their positive attitudes toward financial issues and their stronger future orientation (Donnelly et al., 2012). Since mental accounting is supposed to be the cognitive process underlying money management, it is likely to assume that mental accounting practices are also associated with conscientiousness. The first study addressing this was again the survey on mental accounting of taxes which builds on the findings from the present work. Therein, conscientiousness was correlated with the mental accounting measure. The relation, however, was non-significant when controlling for other variables such as time orientation and impulsivity (Olsen et al., 2019).

Research on correlates of mental accounting is still in its infancy. Prior studies identified some variables and psychological concepts that are related to the mental accounting process. Since most of the scales applied in preceding endeavors to measure mental accounting were specifically developed for the context of managing the tax due, it is unclear whether findings are generalizable to mental accounting of other financial activities. Therefore, a more thorough exploration of individual differences in mental accounting practices and their correlates is needed.

## The Mental Accounting Scale, Research Questions, and Overview of Studies

The present paper uses a Likert-type scale to assess individual differences in mental accounting, i.e., the degree to which mental accounting is applied to keep overview of expenses. The five items of this *mental accounting scale* were loosely based on results from an interview study and previous measures (Soman, 2001; Muehlbacher and Kirchler, 2013) and reflect basic elements from the theory: separating between different categories of financial activities, having good overview, and being well organized. Participants expressed their agreement to each statement on a seven-point scale, ranging from 1 (*strongly disagree*) to 7 (*strongly agree*). The exact wording of the items, their means, and factor loadings can be found in Table 1. In each sample of the three studies (*N* = 165, *N* = 114, and *N* = 373) presented in the following, a principal components analysis yielded a single factor (applying Kaiser’s criterion of an eigenvalue over 1) with eigenvalues of 2.71, 2.39, and 2.55, respectively, explaining 54.21, 47.78, and 50.95% of variance. Reliability of the scale with α = 0.77, α = 0.72, and α = 0.75, respectively, is acceptable.

**TABLE 1:** Items and descriptive characteristics of the mental accounting scale.

	**Study 1**		**Study 2**		**Study 3**	
**Item**	***M* (*SD*)**	**Factor loading**	***M* (*SD*)**	**Factor loading**	***M* (*SD*)**	**Factor loading**
1. It is important to me to keep track of my financial activities precisely	5.99 (1.23)	0.77	6.37 (0.90)	0.59	5.69 (1.54)	0.76
2. I keep a record of my earnings and expenses	3.48 (2.08)	0.81	4.03 (1.92)	0.80	3.44 (2.03)	0.83
3. I could at least say roughly how much I have spent this month	4.97 (1.66)	0.82	5.18 (1.60)	0.76	4.90 (1.76)	0.73
4. I classify my expenses into different categories (e.g., clothing, entertainment, education.)	3.03 (2.06)	0.65	3.40 (1.85)	0.67	2.69 (1.87)	0.57
5. Generally, I am someone others would describe as “well organized”	5.22 (1.58)	0.61	5.13 (1.52)	0.61	4.59 (1.69)	0.66
Scale Mean (*SD*)	4.54 (1.27)		4.82 (1.09)		4.26 (1.26)	
Cronbach’s α	α = 0.77		α = 0.72		α = 0.75	
Eigenvalue	2.71		2.39		2.55	
Explained variance	54.21%		47.78%		50.95%	
*N*	165		114		373	

**1 – strongly disagree to 7 – strongly agree. The number of factors was determined by applying Kaiser’s criterion of an eigenvalue over 1.**

In experimental Studies 1 and 2, the measure is used to test whether individual differences moderate the well-documented effects of keeping mental accounts on spending in decision scenarios involving prior losses (Tversky and Kahneman, 1981) and gains (Fogel, 1997). Such a moderation effect would support our proposition that individual differences in mental accounting matter by affecting the decision process. Further, it would provide evidence for the construct validity of the mental accounting scale.

Second, the scale is applied in a survey among a convenience sample from Austria to explore for correlates of mental accounting. The questionnaire for the survey included the socio-demographic variables age, sex, education, and income, as well as the psychological constructs reviewed in the previous section that were found or hypothesized to be associated with the mental accounting process. These were the Big Five of personality (extraversion, agreeableness, conscientiousness, emotional stability, and openness), the three types of impulsivity (motor impulsivity, non-planning impulsivity, and attentional impulsivity), financial literacy, short-term time orientation, and long-term time orientation. Owing to the scarce and ambiguous prior evidence, the analysis of the survey responses has a purely explorative rationale with the purpose of identifying potential correlates of mental accounting.

## Study 1

The rationale of the first study is to demonstrate that individual differences in mental accounting matter. For this purpose, it will be tested whether scores on our mental accounting scale moderate the effect of the lost theater-ticket problem (Kahneman and Tversky, 1984). The theater-ticket problem was chosen because it is probably the most prominent example of mental accounting and its effect was frequently replicated with slight variations in the design (e.g., Henderson and Peterson, 1992; Heath and Soll, 1996; Chatterjee et al., 2009). A notable characteristic of the two experimental treatments is that they allow us to study the effects of mental accounting when processing prior losses (as opposed to gains). The data and material of the study can be found in the online repository (https://osf.io/r7vgc/).

### Materials and Methods

#### Participants

Participants were recruited via email. Data were collected in 2016. Overall, 167 subjects completed the online questionnaire, but 2 left the dependent variable blank and were deleted from the dataset, resulting in a final sample of *N* = 165. The average age was *M* = 33.90 (*SD* = 10.07) years, 74.2% were female, and 21.8% were employed or self-employed. A rather large part of the sample (36.5%) indicated that they have already known Kahneman and Tversky’s (1984) theater-ticket problem from the media, the literature, or their studies. However, having heard of this decision problem before neither predicted participants’ choices nor interacted with the experimental treatments. Participation in the study was not remunerated.

#### Materials

The online survey started with the five items of the *mental accounting scale* (α = 0.77, *M* = 4.54, *SD* = 1.27, 1 – *strongly disagree*, 7 – *strongly agree*). After completing the scale, the participants were presented with one of the two scenarios of the theater-ticket problem (for the original English version of the instructions, see Kahneman and Tversky, 1984, p. 347) and indicated if they were willing to buy another ticket after noticing the loss of either a pre-bought ticket or a €10 bill. The survey finished with collecting socio-demographic characteristics and the question whether participants have known the theater-ticket problem before the study.

### Results

In general, the percentage of participants willing to buy another theater ticket after noticing the loss of a pre-bought ticket or a banknote of identical value was high (90.9%). As in the original study (Kahneman and Tversky, 1984), willingness to spend further €10 on another ticket was higher in the lost-money condition (97.0%) than in the lost-ticket condition (81.8%), χ^2^(1, *N* = 165) = 11.00, *p* = 0.001. The exact frequencies for both experimental treatments are depicted in Table 2.

**TABLE 2:** Frequencies of choices in the theater-ticket scenario.

		**Lost ticket**	**Lost banknote**
Buy another ticket?	No	12 (18.2%)	3 (3.0%)
	Yes	54 (81.8%)	96 (97.0%)

**N = 165. χ^2^ = 11.00^∗∗∗^, df = 1. Numbers in parentheses indicate column percentages. ^∗∗∗^p = 0.001.**

**FIGURE 1:**
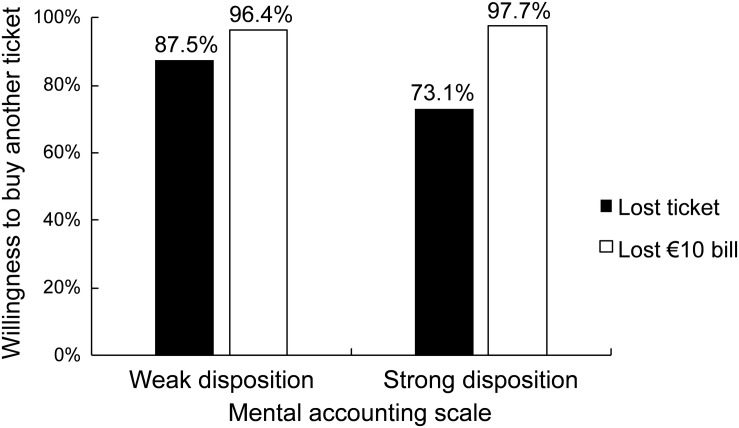
Effect of the theater-ticket scenario for participants with weak and strong dispositions to engage in mental accounting.

The role of individual differences in mental accounting when responding to the lost-ticket scenario was tested by means of logistic regression. Participants’ responses were regressed to experimental treatment (lost ticket vs. lost banknote), the z-transformed scores on the mental accounting scale (1 – *low propensity to engage in mental accounting*; 7 – *high propensity*), and the interaction of both predictors. The results are summarized in Table 3. As expected, the interaction of the lost-ticket scenario and the score on the mental accounting scale predicted participants’ choices. Whether a ticket or a €10 bill was lost matters only for participants with high values in the mental accounting scale. Note, however, that with *b* = -0.58, *SE* = 0.35, *OR* = 0.56, *p* = 0.093, the interaction effect is only marginally significant in Model 1 of the regression analysis. For further exploration of its relation with choices, potential covariates were identified to be included in a second regression model. Since the inclusion of covariates was not planned *a priori*, however, it has to be emphasized that this additional analysis is explorative, and its findings have to be considered preliminary. From the available socio-demographic characteristics, only participants’ age was correlated with the dependent variable, *r*_s_ = 0.18, *p* = 0.034; older participants seem to be more likely to buy a theater ticket regardless of the experimental condition. Including age as an additional predictor (though with *b* = 0.11, *SE* = 0.06, *OR* = 1.12, *p* = 0.054 only marginally significant) reduces the *p*-value of the interaction term to *p* = 0.014 (*b* = -1.53, *SE* = 0.62, *OR* = 0.22, *p* = 0.014; Bonferroni adjusted α = 0.03) and the *p*-value of the effect of the scenario presented to participants to *p* = 0.030 (*b* = -2.01, *SE* = 0.93, *OR* = 0.13, *p* = 0.030; see Model 2 in Table 3). Because many participants left the field for their age blank, the sample size used for the second regression model is reduced to *n* = 139.^2^ Figure 1 summarizes the moderating role of the mental accounting scale for the effect typically observed in Kahneman and Tversky’s (1984) theater-ticket problem. For this depiction, participants were split in two groups based on the median score of the scale (*Mdn* = 4.6). Although the difference in frequencies between the lost-banknote and lost-ticket conditions is small and non-significant for participants with a weak disposition for mental accounting, for those with a strong disposition, the typical choice pattern was observed, which was also found in previous studies. Remarkably, a strong disposition to engage in mental accounting decreases the willingness to buy another ticket after noticing its loss, but – in line with mental accounting theory – does not affect spending after losing a bank note.

**TABLE 3:** Logit regression predicting choices in the theater-ticket scenario.

	**Buy another ticket?^a^**					
	**Model 1**			**Model 2**		
**Variable**	***b***	***SE***	***OR***	***b***	***SE***	***OR***
Constant	2.60	0.38	13.43	0.15	1.76	1.17
Scenario^b^	–1.04	0.38	0.35^∗∗^	–2.01	0.93	0.13^∗^
Mental accounting scale	0.05	0.35	1.05	0.91	0.62	2.48
Scenario × Mental accounting scale	–0.58	0.35	0.56	–1.53	0.62	0.22^∗^
Age				0.11	0.06	1.12^††^
*Nagelkerke R*^2^	0.19			0.37		
χ^2^	14.77^∗∗^			25.81^∗∗∗^		
*n*	165			139		

**Criterion was the choice in the respective scenario. b = unstandardized coefficient; SE = standard errors; OR = dds ratio. Scores on the mental accounting scale were z-transformed. ^*a*^Coded 1 = yes, 0 = no. ^*b*^Coded -1 = lost banknote, 1 = lost ticket. ^†^p = 0.093. ^††^p = 0.054. ^∗^p < 0.05. ^∗∗^p < 0.01. ^∗∗∗^p < 0.001.**

## Study 2

The second study has a similar rationale as Study 1, focusing, however, on the mental organization of income, i.e., the processing of prior gains rather than losses. For this purpose, a scenario that was based on the material from one of the rare studies on this aspect of mental accounting conducted by Fogel (1997) was applied. Her working paper was popularized by Thaler’s seminal review (therein, the paper was cited by using the author’s former surname, O’Curry), but was not published since. Because the original material was not described comprehensively in her manuscript, own scenarios were developed, which were inspired by the prior study. As described in detail in the following section, participants read scenarios describing the reception of money either from a serious source (a tax refund) or from a frivolous source (casino winnings) and ranked six alternative uses of the money, which also varied with respect to their seriousness and frivolousness, respectively. It is expected (i) that – as in Fogel’s original study – money from the serious/frivolous source is more likely spent for serious/frivolous expenses and (ii) that the source of income matters only when dealing with larger amounts of money. For lower amounts, the source should be less relevant in choosing between the different spending opportunities. Further, in line with the rationale of the present research, it is expected that (iii) scores on the mental accounting scale moderate the effects described above.

Originally, Study 2 also included another experimental treatment that was designed to extend the previous research from spending after receiving a prior gain to saving after a loss has occurred. Complementary to the two sources of the gain, the loss was either described to be caused by a serious source (an unexpected additional tax payment) or by a frivolous source (casino losing). Participants in this condition were asked to rank the alternatives in line with their preferences where to save money to compensate for the loss described in the scenario. However, the operationalization in this additional treatment seems to have failed, and the hypothesized source effect was not observed in this condition. Hence, its results are omitted in the following. Results regarding the other conditions remain the same when this additional condition is included in the analysis. The complete data set (with a total *N* = 232), a summary of the analysis of all experimental conditions, and the material of the study can be found in the online repository (https://osf.io/r7vgc/).

### Materials and Methods

#### Participants

Participants were recruited in several courses from different study disciplines. Data were collected in 2012. The paper–pencil questionnaire was completed by *N* = 114 subjects with an average age of *M* = 24.20 (*SD* = 4.85) years. 70.2% were female, 38.6% indicated to have less than €500 available per month, 45.6% have €500–1000 at their disposal, and 15.8% more than that. Participation in the study was not remunerated.

#### Materials

The study was conceptualized as a paper–pencil experiment with a 2 (income source: casino winnings vs. tax refund) by 2 (amount: €25 vs. €250) between-subjects design. Each participant was randomly assigned to one of the overall four experimental conditions. A short scenario introducing the questionnaire manipulated the two factors. In the casino scenarios, participants read that they should imagine having won €25/€250 in a casino. In the tax scenarios, they read that their tax report for this year yielded a refund of €25/€250. On the last page of the experimental questionnaire, the two sources of income described in the scenarios were assessed on two Likert-type items (1 – *boring* to 7 – *exciting* and 1 – *amusing* to 7 – *serious*, recoded), which were combined to form an index ranging from 1 (*serious*) to 7 (*frivolous*). Each participant evaluated both sources regardless of the experimental condition. This frivolousness index was *M* = 6.42, *SD* = 0.82 for the casino winnings and *M* = 3.30, *SD* = 1.78 for the tax refund. A dependent sample t-test confirms that frivolousness of the two income sources was perceived differently, and thus, the manipulation of this factor was successful, *t*(113) = -18.37, *p* < 0.001.

After reading the scenario, the participants had to assign rank numbers from 1 to 6 to several spending opportunities (buying a present for oneself, birthday and Christmas presents, clothing, eating at restaurants, paying the bills, and saving the money) so that each rank stands for the likelihood that the money would be spent at the respective opportunity. The six spending opportunities differed regarding whether they concerned frivolous (i.e., amusing and exciting) or rather serious (i.e., serious and boring) expenses. The grouping of expenses was based on participants’ ratings of each alternative on similar items as applied for evaluating the income sources (1 – *boring* to 7 – *exciting* and 1 – *amusing* to 7 – *serious*, recoded). The evaluation was done after the participants completed the ranking of the six spending opportunities. Table 4 shows the means and standard deviations of the resulting frivolousness index for each spending opportunity. A principal components analysis found three dimensions (applying Kaiser’s criterion of an eigenvalue over 1) underlying the evaluation of the expenses’ frivolousness. The factor loadings obtained by this analysis are depicted in Table 4. As expected, buying a present for oneself, birthday and Christmas presents, and clothing are cumulated on the same dimension, whereas paying the bills and saving the money are on another dimension. Expenses for eating at restaurants, however, have the highest loadings on the third factor. The emergence of this third factor can be explained by previous research on malleable accounting pointing out the ambiguity of restaurant expenses in mental categorization. According to this, expenses for a restaurant visit could be mentally booked as “evening entertainment,” as “costs for food,” or even as “work costs” if it is work-related dinner (Cheema and Soman, 2006; Zhang and Sussman, 2018). Owing to the ambiguous evaluation of restaurant expenses also in the present study, this spending category was excluded from the analysis. Hence, three of the spending opportunities were considered frivolous expenses (present for oneself, birthday and Christmas presents, and clothing), and two as serious expenses (paying the bills and savings). Two ranking scores were computed for these two spending categories by averaging the rank numbers assigned to the expenses in each category. The mean rank for the frivolous spending category was *M* = 3.47, *SD* = 0.71, and for the serious spending category, it was *M* = 3.25, *SD* = 1.19. Next, these two means were subtracted from each other to form a single index ranging from -3.5 to + 3.5, with negative values indicating that the money described in the scenario is more likely to be spent on frivolous expenses and positive values indicating a higher likelihood for serious expenses. The resulting *spending category index* (*M* = 0.22, *SD* = 1.79, -3.5 – *frivolous expenses* to + 3.5 – *serious expenses*) serves as a dependent variable in the analyses presented in the following.

**TABLE 4:** Principal component analysis of evaluations of spending opportunities (after varimax rotation).

	***M*^a^**	***SD*^a^**	**F1^a^**	**F2^b^**	**F3^c^**
Present for oneself	5.93	1.04	0.01	0.60	0.11
Clothing	5.61	1.11	−0.23	0.71	0.10
Birthday/Christmas presents	5.36	0.98	0.15	0.70	−0.26
Eating at restaurants	4.10	1.18	0.04	0.04	0.96
Savings	2.61	1.33	0.83	0.09	−0.08
Paying the bills	1.96	1.00	0.79	−0.13	0.11

**N = 114. Alternatives were rated on two items (1 – boring to 7 – exciting and 1 – amusing to 7 – serious, recoded) to form an index from 1 (serious) to 7 (frivolous). The number of factors was determined by applying Kaiser’s criterion of an eigenvalue over 1. ^*a*^Eigenvalue = 1.46, 24.38% explained variance. ^*b*^Eigenvalue = 1.30, 21.68% explained variance. ^*c*^Eigenvalue = 1.02, 17.01% explained variance.**

Finally, before socio-demographic characteristics were collected, participants responded to the five items of the *mental accounting scale* (α = 0.72, *M* = 4.82, *SD* = 1.09, 1 – *strongly disagree* to 7 – *strongly agree*).

### Results

The average ranks assigned to each of the six spending opportunities are shown in Table 5. Across the experimental conditions, the most likely options the income is spent for were buying clothing and paying the bills. By contrast, the options with the lowest ranks, i.e., the least likely opportunities, were buying birthday/Christmas presents and savings (and eating at restaurants, which was excluded from the analysis). As shown in the lower part of Table 5, the averaged ranks per spending category were about the same for the frivolous expenses and the serious expenses, *t*(113) = 1.31, *p* = 0.194. Accordingly, the spending category index (ranging from -3.5 – *frivolous expenses* to + 3.5 – *serious expenses*) with *M* = 0.22, *SD* = 1.79 was close to zero.

**TABLE 5:** Means and standard deviations of the ranking of spending opportunities and of the spending category index by experimental condition.

	**Experimental condition**				
	**Tax refund**		**Casino winnings**		
	**€25**	**€250**	**€25**	**€250**	**Total**
	**(*n* = 31)**	**(*n* = 29)**	**(*n* = 26)**	**(*n* = 28)**	**(*N* = 114)**
	***M* (*SD*)**	***M* (*SD*)**	***M* (*SD*)**	***M* (*SD*)**	***M* (*SD*)**
Present for oneself	3.32 (1.40)	3.48 (1.50)	3.35 (1.98)	2.93 (1.39)	3.27 (1.56)
Birthday/Christmas presents	4.23 (1.43)	3.93 (1.13)	4.19 (1.58)	4.04 (1.29)	4.10 (1.35)
Clothing	3.16 (1.19)	3.55 (1.64)	2.73 (1.25)	2.61 (1.23)	3.03 (1.37)
Eating at restaurants	3.84 (1.81)	4.41 (1.52)	3.77 (1.53)	4.43 (1.26)	4.11 (1.56)
Savings	3.81 (1.92)	3.24 (1.77)	3.42 (1.65)	3.29 (2.00)	3.45 (1.83)
Paying the bills	2.65 (2.01)	2.38 (2.01)	3.54 (1.98)	3.71 (2.24)	3.04 (2.11)
Frivolous expenses^a^	3.57 (0.77)	3.66 (0.80)	3.42 (0.59)	3.19 (0.58)	3.46 (0.71)
Serious expenses^b^	3.23 (1.22)	2.81 (1.32)	3.48 (1.09)	3.50 (1.01)	3.25 (1.19)
Spending category index^c^	0.34 (1.85)	0.84 (2.03)	−0.06 (1.56)	−0.31 (1.51)	0.22 (1.79)

**N = 114. Ranks ranged from 1 (most likely) to 6 (least likely). ^*a*^Frivolous expenses = present for oneself, birthday/Christmas presents, and clothing. ^*b*^Serious expenses = savings and paying the bills. ^*c*^Spending category index = mean rank of frivolous expenses – mean rank of serious expenses.**

To test hypotheses (i) and (ii), a linear regression was estimated, with spending category index as a dependent variable and the two manipulated factors (source: casino vs. tax; amount: €25 vs. €250) as predictors. The results of this analysis are summarized in the left panel (Model 1) of Table 6. As expected, the spending category was significantly affected by income source, *b* = -0.39, *SE* = 0.17, *p* = 0.020. Although casino winnings were more likely to be spent on frivolous expenses, money from a tax refund was more likely to be spent on serious expenses. In contrast to Fogel’s (1997) previous study, no interaction of the amount with the source of income was observed in the first regression model. The amount of the income described in the scenarios did not affect decisions. Note, however, that the omnibus test for the first regression model with *F*(3, 110) = 2.33, *p* = 0.078 is only marginally significant.

**TABLE 6:** OLS regression predicting spending category index by experimental condition and mental accounting disposition.

	**Spending category index**					
	**Model 1**			**Model 2**		
**Variable**	***b***	***SE***	**β**	***b***	***SE***	**β**
Constant	0.21	0.17		0.15	0.16	
Source^a^	–0.39	0.17	−0.22^∗^	–0.43	0.16	–0.24^∗∗^
Amount^b^	0.06	0.17	0.04	–0.08	0.16	0.05
Source × Amount	–0.19	0.17	–0.11	–0.17	0.16	–0.10
Mental accounting scale				0.39	0.16	0.22^∗^
Source × Mental accounting scale				–0.15	0.16	–0.08
Amount × Mental accounting scale				–0.06	0.16	–0.03
Source × Amount × Mental accounting scale				–0.37	0.16	−0.21^∗^
*R*^2^		0.06			0.15	
*F*		2.33^†^			2.73^∗^	
Δ*R*^2^					0.09	
Δ*F*					2.91^∗^	

**N = 114. Criterion was the spending category index ranging from -3.5 (money is spent on frivolous expenses) to + 3.5 (money is spent on serious expenses). Scores on the mental accounting scale were z-transformed. ^*a*^Coded -1 = tax, + 1 = casino. ^*b*^Coded -1 = €25, + 1 = €250. ^†^p = 0.078. ^∗^p ≤ 0.05. ^∗∗^p ≤ 0.01.**

**FIGURE 2:**
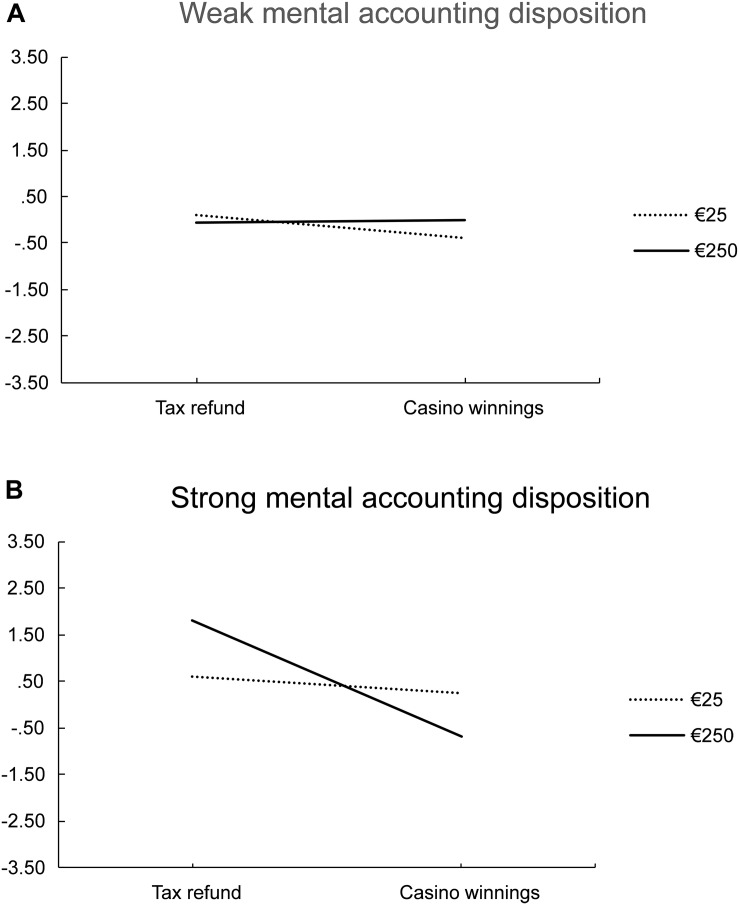
**(A,B)** Interaction effect between income source, amount of income, and mental accounting disposition. The spending category index indicates which type of expenses the money is more likely to be spent on, ranging from -3.5 (frivolous expenses) to + 3.5 (serious expenses).

To test hypothesis (iii) that individual differences in mental accounting moderate the effect of income source, a second regression was estimated. In Model 2, the z-transformed scores on the mental accounting scale and its interactions with the experimental treatments were added as predictors. A summary of this analysis is shown in the right panel (Model 2) of Table 6. When controlling for individual differences in mental accounting, a similar effect of income source was observed as in the first model, *b* = -0.43, *SE* = 0.16, *p* = 0.010. Scores on the mental accounting scale were positively related to the spending category index, i.e., a strong disposition to engage in mental accounting led to spending the income more on serious expenses, *b* = 0.39, *SE* = 0.16, *p* = 0.020. Further, a three-way interaction of income source, amount of income, and individual differences in mental accounting was observed, *b* = -0.37, *SE* = 0.16, *p* = 0.026. The means of the spending category index resulting from this interaction are shown in Figures 2A,B. For this depiction, participants were split into two groups by the median of the mental accounting scale (*Mdn* = 4.80). Although for participants with a weak mental accounting disposition (Figure 2A) the source and amount of income did not matter when spending the money, participants with a strong disposition (Figure 2B) were more likely to spend money from a frivolous source for frivolous expenses and money from a serious source for serious expenses, but only if the amount of income was high. For smaller amounts, also in this group, the source of income did not affect spending.^3^

## Study 3

After testing the moderating effects of individual differences in mental accounting, in Study 3, the mental accounting measure was applied in a questionnaire survey of a convenience sample from Austria. The motivation for this survey was to explore for correlates of mental accounting. The first set of variables collected was the participants’ socio-demographic characteristics (age, sex, education, and income). The second regarded the Big Five of personality (extraversion, agreeableness, conscientiousness, emotional stability, and openness). The role of the five traits for mental accounting was explored because of findings from research on money management, a construct closely related to mental accounting. The third set of measures concerned the three types of impulsivity (motor impulsivity, non-planning impulsivity, and attentional impulsivity) described in the theory section. Impulsivity was included in the exploration for correlates because mental accounting is assumed to be a self-control tool used predominantly by rather non-impulsive individuals. Further, inspired by previous findings on correlates of mental accounting, financial literacy was tested in the survey, and participants’ short-term and long-term time orientations were measured. Note that the analyses presented in the following have a purely explorative rationale, and its findings should therefore be considered preliminary and need to be replicated in future studies. The data and material of the study can be found in the online repository (https://osf.io/r7vgc/).

### Materials and Methods

#### Participants

A sample of *N* = 373 subjects was recruited in several small businesses who allowed their employees to participate in the study. Data were collected in 2014. Participants were *M* = 25.22 (*SD* = 10.21) years old, 31.6% were female, and they indicated earning a monthly net income of *M* = 1023.19 (*SD* = 728.35). 65 participants did not respond to the question about income, and for the analysis presented in the following, missing values were replaced with the sample mean.^4^ Regarding the level of education, 3.8% indicated to have compulsory education, 30.3% had a vocational training, 56.3% held the general qualification for university entrance, and 9.7% had an academic education. Participation in the study was not remunerated.

#### Materials

The paper–pencil questionnaire started with questions about socio-demographic characteristics. Then, a short version of the Big Five Inventory (Rammstedt and John, 2007) captured respondents’ personality on five dimensions (*extraversion*, *agreeableness*, *conscientiousness*, *neuroticism*, and *openness*) by a seven-point Likert-type scale consisting of 10 items.^5^ Explorative factor analysis of these 10 items yielded the expected five dimensions (applying Kaiser’s criterion of an eigenvalue over 1). The first of the two items regarding agreeableness, however, loaded equally high on a second factor, that is, openness. Accordingly, except for agreeableness, all correlations between the two corresponding items per dimension were substantial enough to be combined to a single measure for each of the traits (extraversion: *r* = 0.47, *p* < 0.001; agreeableness: *r* = 0.04, *p* = 0.503; conscientiousness: *r* = 0.25, *p* < 0.001; neuroticism: *r* = 0.31, *p* < 0.001; and openness: *r* = 0.37, *p* < 0.001). Thus, for the analysis of agreeableness, only one of the two items will be used. The second item was chosen because it unambiguously loaded only on the respective dimension.^6^ The other items were averaged pairwise to composite indices for the remaining four dimensions of personality (1 – *low value on the respective dimension*; 7 – *high value on the respective dimension*). Means and standard deviations for the five personality measures, as well as their intercorrelations, are shown in Table 7.

**TABLE 7:** Intercorrelations (Pearson) of measures in Study 3.

**Variable**	**n**	***M* (*SD*)**	**1**	**2**	**3**	**4**	**5**	**6**	**7**	**8**	**9**	**10**	**11**	**12**	**13**	**14**	**15**
1. Mental accounting scale	373	4.26(1.26)	–														
2. Age	373	25.22(10.20)	0.13^∗^	–													
3. Sex^a^	373	0.32	0.08	0.35^∗∗∗^	–												
4. Education^b^	373	2.72(0.69)	–0.09	0.00	0.15^∗∗∗^	–											
5. Income	308	1023.19(728.35)	0.03	0.49^∗∗∗^	0.12^∗^	0.07	–										
6. Personality: Extraversion	372	4.9(1.28)	0.01	–0.19^∗∗∗^	0.00	0.04	−0.13^∗^	–									
7. Personality: Agreeableness	372	4.4(1.59)	–0.01	0.00	0.00	−0.13^∗^	–0.08	–0.03	–								
8. Personality: Conscientiousness	372	5.13(1.22)	0.41^∗∗∗^	0.24^∗∗∗^	0.16^∗∗∗^	0.02	0.15^∗∗^	0.15^∗∗∗^	0.03	–							
9. Personality: Neuroticism	372	3.5(1.29)	−0.11^∗^	0.07	0.19^∗∗∗^	–0.07	0.06	–0.27^∗∗^	–0.08	–0.20^∗∗^	–						
10. Personality: Openness	372	4.67(1.49)	0.02	0.05	0.23^∗∗∗^	0.12^∗^	–0.03	0.13^∗^	0.02	0.09	–0.08	–					
11. Motor impulsivity	372	4.07(1.07)	–0.19^∗∗∗^	–0.21^∗∗∗^	0.01	0.06	–0.09	0.25^∗∗^	–0.07	–0.16^∗∗^	−0.12^∗^	0.08	–				
12. Non-planning impulsivity	372	3.01(1.09)	–0.47^∗∗∗^	0.01	0.10	0.04	0.04	−0.13^∗^	0.02	–0.37^∗∗∗^	0.09	–0.04	0.26^∗∗∗^	–			
13. Attentional impulsivity	372	3.12(1.01)	–0.17^∗∗∗^	–0.03	0.01	–0.07	0.07	–0.08	−0.11^∗^	–0.29^∗∗∗^	0.28^∗∗∗^	−0.13^∗^	0.24^∗∗∗^	0.26^∗∗∗^	–		
14. Financial literacy	373	2.79(1.34)	0.12^∗^	0.07	–0.19^∗∗∗^	0.19^∗∗∗^	0.04	–0.04	–0.03	0.01	−0.12^∗^	0.02	–0.03	–0.03	–0.09	–	
15. Short-term time orientation	373	3.41(1.13)	–0.25^∗∗∗^	–0.05	–0.01	–0.02	0.02	–0.05	–0.02	–0.29^∗∗^	0.07	–0.05	0.25^∗∗∗^	0.37^∗∗∗^	0.22^∗∗∗^	–0.06	–
16. Long-term time orientation	373	4.82(1.07)	0.33^∗∗∗^	–0.02	−0.13^∗^	0.01	0.02		–0.13^∗∗^	0.19^∗∗∗^	–0.03	0.09	–0.28^∗∗∗^	–0.61^∗∗∗^	–0.17^∗∗∗^	0.08	–0.36^∗∗∗^

**N = 373. All variables, except sex, education, and income, were measured on a Likert-type scale with values from 1 (low) to 7 (high). ^*a*^Coded 0 = male, 1 = female. ^*b*^Coded as 1 = compulsory education, 2 = vocational training, 3 = general qualification for university entrance, and 4 = academic education. ^∗^p ≤ 0.05. ^∗∗^p ≤ 0.01. ^∗∗∗^p ≤ 0.001.**

The Big Five Inventory was followed by the items of the *mental accounting scale* (α = 0.75, *M* = 4.26, *SD* = 1.26, 1 – *strongly disagree* to 7 – *strongly agree*).

Next in the questionnaire, 15 Likert-type items measured participants’ impulsivity. The German short version of the Barratt Impulsiveness Scale BIS-15 (Meule et al., 2011) was applied to assess the degree of impulsiveness on three 7-point subscales (1 – *low impulsivity*, 7 – *high impulsivity*). Explorative factor analysis found the expected three dimensions (on basis of the scree-plot, applying Kaiser’s criterion would suggest a fourth factor with an eigenvalue of 1.04), each consisting of five items: *non-planning* (α = 0.84), *motor* (α = 0.75), and *attentional impulsivity* (α = 0.64). Table 7 shows the means and standard deviations for the three types of impulsivity, as well as their intercorrelations.

Financial literacy was assessed by the so-called Big Five questions (Hastings et al., 2013), testing participants’ knowledge and understanding of fundamental concepts in finance such as compound interest, real rates of return, risk diversification, mortgage interest, and bond prices (see Table 1, p. 353 in Hastings et al., 2013 for the wording of the five questions). Correct answers to the five questions were summed to a *financial literacy* score ranging from 0 (*low*) to 5 (*high*) (*M* = 2.79, *SD* = 1.34).

The last scales in the questionnaire concerned long-term and short-term time orientations. The items for these scales were taken from Antonides et al. (2011; see Appendix A therein for the wording of the items). The four items of the *short-term time orientation* scale (α = 0.62, *M* = 3.41, *SD* = 1.13, 1 – *low*, 7 – *high*) measure the extent of focusing on the short term, neglecting the future, and evaluating the importance of the present. The *long-term time orientation* scale (α = 0.65, *M* = 4.83, *SD* = 1.07, 1 – *low*, 7 – *high*) consists of four items and captures respondents’ propensity to take care of the future, to make long-term investments, to save money, and to take precautions for harder times.

### Results

The means, standard deviations, and zero-order correlations for all variables are shown in Table 7. Values on the mental accounting scale were correlated with 9 of the 15 variables. Multiple linear regression was used to examine these relations while controlling for all measures simultaneously. Table 8 summarizes the results from this analysis. Regarding respondents’ socio-demographic variables, as in prior research (Antonides et al., 2011), being female, *b* = 0.42, *SE* = 0.14, *p* = 0.003, and having a lower education level, *b* = -0.17, *SE* = 0.06, *p* = 0.004, were related with higher scores on the mental accounting scale. Regarding the personality traits, engaging in mental accounting was positively related to conscientiousness, *b* = 0.32, *SE* = 0.07, *p* < 0.001, and to low non-planning impulsivity, *b* = -0.41, *SE* = 0.08, *p* < 0.001. In contrast to prior findings (Antonides et al., 2011), short-term time orientation and long-term orientation were not significantly related with mental accounting in the regression analysis. Note, however, that in the zero-order correlation analysis presented in Table 7, both measures for time orientation were associated with mental accounting and with the three types of impulsivity, particularly with non-planning impulsivity. This could mean a complex interrelation between impulsivity, time orientation, and mental accounting. Non-planning impulsivity might, for instance, mediate the relation of short-term orientation and mental accounting. Regarding financial literacy, engaging in mental accounting was associated with higher financial knowledge, *b* = 0.18, *SE* = 0.06, *p* = 0.002, in the regression analysis presented in Table 8.

**TABLE 8:** Exploration for correlates of the mental accounting disposition by OLS regression.

	**Mental accounting scale**		
**Variable**	***b***	***SE***	**β**
Constant	4.13	0.07	^∗∗∗^
Age	0.00	0.07	0.00
Sex^a^	0.42	0.14	0.15^∗∗^
Education^b^	–0.17	0.06	–0.13^∗∗^
Income^c^	–0.03	0.06	–0.02
Personality: Extraversion^d^	–0.09	0.06	–0.07
Personality: Agreeableness^d^	–0.04	0.06	–0.03
Personality: Conscientiousness^d^	0.32	0.07	0.25^∗∗∗^
Personality: Neuroticism^d^	–0.11	0.06	–0.08
Personality: Openness^d^	–0.05	0.06	–0.04
Motor impulsivity^d^	–0.03	0.06	–0.03
Non-planning impulsivity^d^	–0.41	0.08	–0.33^∗∗∗^
Attentional impulsivity^d^	0.03	0.06	0.03
Financial literacy	0.18	0.06	0.15^∗∗^
Short-term time orientation	–0.03	0.06	–0.03
Long-term time orientation	0.09	0.07	0.07

**N = 373, R^2^ = 0.34, F(15, 372) = 12.07^∗∗∗^. All tolerance values were greater than 0.55. The criterion was the score on the mental accounting scale. All variables, except age, sex, education, and income, were measured on a Likert-type scale with values from 1 (low) to 7 (high). All non-dichotomous predictors are z-transformed. ^*a*^Coded 0 = male, 1 = female. ^*b*^Coded as 1 = compulsory education, 2 = vocational training, 3 = general qualification for university entrance, and 4 = academic education. ^*c*^65 missing values were replaced by the sample mean. ^*d*^One missing value was replaced by the sample mean. ^∗∗^p ≤ 0.01. ^∗∗∗^p ≤ 0.001.**

## General Discussion

Mental accounting is a theory about the cognitive processes underlying money management and financial decision-making. Individual differences in mental accounting have rarely been studied in prior research, and little is known about correlated variables and concepts. In the three studies reported here, a five-item Likert-type scale was applied to measure individual differences in mental accounting, i.e., the extent individuals engage in specific mental accounting practices. In two experiments, participants’ scores on the mental accounting scale moderated the effects of the experimental treatments, which provides evidence for the significance of considering such differences when studying mental accounting. Furthermore, our explorative survey suggests that engaging in mental accounting is correlated with being female, having lower education but good financial literacy, being conscientious, and having low non-planning impulsivity.

The general effects observed in Studies 1 and 2 are in line with previous research. The first experiment replicates prior findings for the lost theater-ticket problem (e.g., Tversky and Kahneman, 1981; Henderson and Peterson, 1992; Heath and Soll, 1996; Chatterjee et al., 2009) and shows that the source of a loss affects spending. In the second experiment, similar mental accounting effects were found regarding the source of a gain. As in the replicated study (Fogel, 1997), income from a serious source was more likely spent for serious expenses than for frivolous expenses. However, a general interaction effect of income source and amount of income as reported in the original study was not found. Instead, we observed a three-way interaction between the amount of income, its source, and the scores on the mental accounting scale. This suggests that only participants with a strong disposition to engage in mental accounting differentiate between income source in their consumption choice, and they do this only if the amount of income is substantial. The moderation effects of the scores on our mental accounting measure observed in Studies 1 and 2 are in line with our hypothesis that individual differences in mental accounting matter and provide evidence for the construct validity of the scale.

The exploration for correlates of individual differences in mental accounting adds to previous research. Regarding the socio-demographic variables, the evidence from the present and from prior studies suggests that mental accounting is pursued more among the less-educated and among females, though the latter relation is less clear. More research is needed to clarify the role of age, income, and wealth in mental accounting because the findings that have been reported so far are inconclusive. In part, the puzzling evidence regarding these variables can be explained by their natural intercorrelation and – regarding the present research – by the relatively low average and little variance of income and age of participants in Study 3, which perhaps has caused the null-correlations observed.

Another group of variables and constructs that were explored concerned respondents’ financial knowledge, impulsivity, and time orientation. As in most of the prior studies, financial knowledge (as captured by five items testing financial literacy; Hastings et al., 2013) was positively related with engaging in mental accounting. An originality of the present research concerns the relation of mental accounting and impulsivity. All three types of impulsivity (i.e., non-planning, motor, and attentional impulsivity) were negatively related with mental accounting in an analysis of zero-order correlations. However, evaluating the impulsivity measures simultaneously in a regression analysis revealed a significant relation solely for the subtype of non-planning impulsivity. Further, in the present study, impulsivity was linked with respondents’ time orientation as to be expected in the zero-order correlations. Being oriented toward the near future was positively associated with impulsivity, and a long-term time orientation was negatively related. Accordingly and in line with prior findings (Antonides et al., 2011; Olsen et al., 2019), short-term time orientation was negatively correlated with mental accounting, whereas long-term orientation was positively related. However, when controlling for the three types of impulsivity in the regression analysis, short and long-term time orientations lose its significance. This could mean a mediating relationship between the two concepts. Short-term time orientation could result in higher impulsivity (or vice versa), leading to less mental accounting. Together, the findings for impulsivity and time orientation provide some support for the notion that mental accounting is a theory about self-control. However, further studies should clarify causalities, i.e., whether mental accounting is mainly applied by less impulsive individuals or whether it leads to less impulsivity by serving as a self-control device.

Another novel aspect of the present research was to link mental accounting to the Big Five of personality traits. Although a small negative relation of neuroticism was also found in the analysis of zero-order correlations, in the regression analysis, only conscientiousness was significantly associated with the mental accounting measure. For conscientiousness, a similar correlation was observed in a taxpayer survey conducted after the present research (however, there it lost significance in a regression analysis controlling also for other variables; see Olsen et al., 2019 for details). In previous research, neuroticism was found to be related with an intuitive decision style, and emotional stability and conscientiousness were related with a deliberate style (Betsch, 2004). Further, these two personality traits were reported to be related with savings, debt, and money management (Brandstätter, 1996, as cited in Pinjisakikool, 2018; Nyhus and Webley, 2001; Mayfield et al., 2008; Donnelly et al., 2012). In sum, the correlations between the different constructs could mean that mental accounting is the product of a non-intuitive, thorough decision process of particularly conscientious individuals and that it positively affects the management of money. Though it is too early to draw such theoretical conclusions from existing evidence, it is surprising that only little prior research considered potential positive effects of mental accounting (notable exceptions regard, for instance, having overview of expenses, Antonides et al., 2011; spending child benefits for the intended purpose, Kooreman, 2000; enhanced tax compliance, Muehlbacher and Kirchler, 2013; and happiness, Sul et al., 2013). Instead, most studies deal with the irrational and negative consequences of the mental accounting process. Future research should therefore address positive effects of mental accounting, such as its self-control function in money management, saving, and spending, and its effects on financial well-being (cf., Zhang and Sussman, 2018).

Limitations of the three studies presented here regard the use of non-representative and rather small convenience samples, and the explorative nature of the survey in Study 3. No clear hypotheses for the surveyed variables were formulated because of the sparse prior evidence. Findings should be replicated in further research employing larger and representative samples of respondents. Further limiting to the present results is the possibility of a common-methods bias as the measures applied in our studies rely on self-reports and hypothetical choices. Another limitation lies in the contents of the Likert-type scale applied to measure the individual extent of engaging in mental accounting. Although the moderation effects observed in the first two studies provide evidence that the measure captures at least some of the relevant features of mental accounting, the items of the scale cover only a few of the aspects that are described in the theory. These regard mainly the mental budgeting process and the resulting better overview of financial activities. Not captured by the scale are, for instance, other mental accounting functions such as simplifying decisions and maximizing pleasure through the principles of hedonic editing. Also not captured by the mental accounting scale are temporal aspects, such as how often balances are checked for instance. Future research could extend the exploration of individual differences also to these and other facets of mental accounting. Moreover, the mental accounting scale used in the present study could be contrasted with existing scales for money management (e.g., Donnelly et al., 2012) in further surveys. Such research would allow us to systematically disentangle the two constructs and to study their relations.

Differences in the quality and extent of mental accounting and their correlated constructs have largely been neglected in previous studies. Future research and theory development would profit from taking individual dispositions into account and from continuing to explore the determinants and consequences of mental accounting. Practical implications of our findings regard, for instance, the need for assistance in efficiently processing financial activities. Providers of financial services should recognize the different dispositions of their clients and encourage favorable mental accounting practices. This could be achieved in training courses or by thought-out decision designs for the choice options presented to customers (as proposed in the nudging theory by Thaler and Sunstein, 2008). An example for such a decision aid was previously implemented in a lab experiment on tax compliance. Participants had to calculate the tax due on their own, or it was automatically computed and indicated on the computer screen. The computerized assistance was particularly helpful for participants with a weak disposition to engage in mental accounting. Without the aid, this group tended to spend too much of their gross income and, consequently, was more prone to evading taxes (Muehlbacher et al., 2017).

Though some of the behavioral consequences of mental accounting may be interpreted as irrational in terms of economic theory, individuals seem to apply its principles to prevent impulsive spending, to keep track of financial activities, and to facilitate money management. The extent to which mental accounting is used as a means of self-control, however, varies. It seems that “mental accounting matters” (Thaler, 1999, p. 183), indeed, but not for everyone to the same degree.

## Data Availability Statement

All datasets generated for this study are included in the article/supplementary material.

## Ethics Statement

Ethical review and approval was not required for the study on human participants in accordance with the local legislation and institutional requirements. Written informed consent from the participants was not required to participate in this study in accordance with the national legislation and the institutional requirements.

## Author Contributions

SM and EK planned the three studies presented in this article. SM collected and analyzed the data. The report was written by SM and corrected by EK.

## Conflict of Interest

The authors declare that the research was conducted in the absence of any commercial or financial relationships that could be construed as a potential conflict of interest.

## Footnotes

1 See Rammstedt and John (2007) for the wording of Likert-type items to measure the Big Five personality traits that were also applied in Study 3.

2 Replacing the 26 missing values for participants age with the sample mean yields *p* = 0.071 for the interaction of the scenario and mental accounting and *p* = 0.011 for the effect of the scenario.

3 A summary of three additional explorative regression analyses with the mean ranks of the frivolous spending category, of the serious spending category, and the rank of the restaurant option (the third category of expenses) can be found in the online repository (https://osf.io/r7vgc/). Results are very similar to the main analysis but seem to suggest that the three-way interaction of income source, amount of income, and scores on the mental accounting scale is mainly driven by differences regarding serious expenses. For the frivolous category alone, only a two-way interaction of income source and scores on the mental accounting scale was found in the additional analysis. Further, for restaurant expenses, a different three-way interaction was found, with changes due to income source only if the amount of income was small and no differences if the amount was large.

4 Excluding participants with missing values from the analysis produced the same results.

5 As one of the reviewers of this paper has pointed out, item 5 of the mental accounting scale (see Table 1) could also be considered an alternative measure for conscientiousness. Excluding this item from the scale and re-running the analyses of all three studies, however, produce exactly the same results.

6 Identical results were obtained when replacing the second item on agreeableness in the regression analysis presented in Table 8 with the first item or with the averaged score of both items.
